# Fascia iliaca compartment block as a preoperative analgesic in elderly patients with hip fractures – effects on cognition

**DOI:** 10.1186/s12877-019-1266-0

**Published:** 2019-09-11

**Authors:** Pär Wennberg, Margareta Möller, Johan Herlitz, Elisabeth Kenne Sarenmalm

**Affiliations:** 1 Research and Development Centre, Skaraborg Hospital, Skövde, Sweden; 2 University Health Care Research Center, Region Örebro and School of Health and Medical Sciences, Örebro University, Örebro, Sweden; 3 Prehospen-Centre of Prehospital Research; Faculty of Caring Science, Work-Life and Social Welfare, University of Borås, Borås, Sweden; 4 Institute of Health and Care Sciences and Centre for Person-Centred Care, and Institute of Health and Care Sciences, Sahlgrenska Academy at the University of Gothenburg, Gothenburg, Sweden

**Keywords:** Hip fractures, Cognitive impairment, Cognitive status, Pain, Nerve block, Pain management, Perioperative care

## Abstract

**Background:**

Impaired cognition is a major risk factor for perioperative delirium. It is essential to provide good pain control in patients with hip fractures and especially important in patients with severely impaired cognitive status, as they receive less pain medication, have poorer mobility, poorer quality of life and higher mortality than patients with intact cognition. The purpose of this study was to examine the association between preoperative pain management with nerve blocks and cognitive status in patients with hip fractures during the perioperative period.

**Methods:**

One hundred and twenty-seven patients with hip fractures participating in a double-blind, randomised, controlled trial were included in this study. At hospital admission, a low-dose fascia iliaca compartment block (FICB) was administered as a supplement to regular analgesia. Cognitive status was registered on arrival at hospital before FICB and on the first postoperative day using the Short Portable Mental Status Questionnaire.

**Results:**

Changes in cognitive status from arrival at hospital to the first postoperative day showed a positive, albeit not significant, trend in favour of the intervention group. The results also showed that patients with no or a moderate cognitive impairment received 50% more prehospital pain medication than patients with a severe cognitive impairment. FICB was well tolerated in patients with hip fractures.

**Conclusion:**

Fascia iliaca compartment block given to patients with hip fractures did not affect cognitive status in this study. Patients with a cognitive impairment may receive inadequate pain relief after hip fracture and this discrimination needs to be addressed in further studies.

**Trial registration:**

EudraCT number 2008–004303-59 date of registration: 2008-10-24.

## Background

Globally, emergency healthcare faces a substantial increase in patients with hip fractures. Estimates indicate that, by 2050, there will there be six million patients with hip fractures annually [1]. In Sweden, 18,000 patients sustain a hip fracture each year. One third of these patients may develop perioperative delirium. A complication of this kind can be significantly reduced using a multi-factorial intervention programme [2]. Hip fracture is a major trauma for the patient and pain management is a challenging task for emergency healthcare, as these patients suffer severe pain [3]. Effective pain treatment requires adequate pain assessment and pain assessment is especially challenging in patients with a cognitive impairment [4].

Delirium is one of the most common perioperative complications in patients with a fractured hip. The reported incidence of perioperative delirium in patients with a hip fracture ranges from 38 to 62% and increases with age, comorbidity and reduced preoperative cognitive status [5, 6]. Furthermore, reduced cognitive status is an independent risk factor for the development of delirium in patients with hip fractures [7, 8]. Cognitive status is defined as a person’s behavioural and cognitive function [9]. Cognitive impairment is defined as a disturbance in the patient’s mental processes related to thinking, reasoning and judgement [10]. This can be manifested as a diagnosis of dementia or as delirium (a state of acute confusion) [11]. These conditions often co-exist. The risk of developing delirium also increases in the presence of dementia [12] and cognitive status should therefore be screened in these patients [13]. Dementia is furthermore an independent risk factor for falling, which puts patients with dementia at increased risk of hip fracture [14].

Studies show that the clinical consequence for patients with hip fractures and cognitive impairment is that they have more pain than lucid patients, because they wait longer for pain relief and receive less than half the pain medication administered to cognitively intact patients [15, 16]. Patients who develop perioperative delirium have more hallucinations and impaired recollection of events [17, 18]. Patients with impaired cognitive status also have a poorer short-term and long-term postoperative outcome after hip fracture than patients with intact cognition [19]. In this case, outcome is defined as a decrease in postoperative recovery relating to activities of daily living (ADL), quality of life and mobility [20], increased length of hospital stay and mortality [21]. The perioperative period is defined as the time periods immediately before, during and following a surgical operation [22].

The risk of delirium increases with limited treatment of pain, so it is essential to provide good pain management [23]. Intermittent fascia iliaca compartment block (FICB) may reduce the incidence of delirium in patients with an intermediate risk of delirium [24] and preoperative FICB may also improve postoperative cognitive status [25]. Research indicates that nerve block is a good choice for pain control, especially in patients with cognitive impairment, as they have reduced abilities to describe their pain and their need for analgesia [26].

The purpose of this study was to examine the association between preoperative pain management with nerve blocks and cognitive status in patients with hip fractures during the perioperative period. The primary aim was to examine the impact of preoperative FICB on cognitive status until the first postoperative day in patients with hip fractures. The secondary aim was to investigate the association between cognitive status and the amount of analgesia given in the preoperative phase of hip fracture care. The hypothesis was that FICB would have a positive impact on cognitive status in these patients as a result of improved pain management.

## Methods

### Design

The patients in this study were participants in a double-blind, randomised, controlled trial [27]. In brief, the purpose of this randomised, controlled trial was to evaluate preoperative pain management with FICB in patients with hip fractures. Patients in the intervention group received an FICB injection with ropivacaine and control patients received an FICB injection with a placebo substance. Fascia iliaca compartment block was added to regular analgesia, i.e. intravenous morphine and paracetamol.

The present study evaluated the perioperative effect of preoperative FICB on cognitive status in patients with a hip fracture.

### Inclusion and exclusion criteria

Patients aged 65 years or more were included consecutively after hospital admission, from October 2010 to February 2012. The inclusion criteria were: 1) patients with a single radiographically confirmed hip fracture and 2) FICB had to be administered less than one hour after hospital admission. The exclusion criteria were 1) multi-trauma; 2) fracture more than 12 h prior to FICB; 3) allergy to local anaesthetics and 4) infection in the injection area. Patients gave their written consent. Those patients who were unable to give their consent were included following presumed consent. Ethical approval and presumed consent were obtained from the Regional Ethics Board in Uppsala.

### Intervention and data collection

**Fig. 1:**
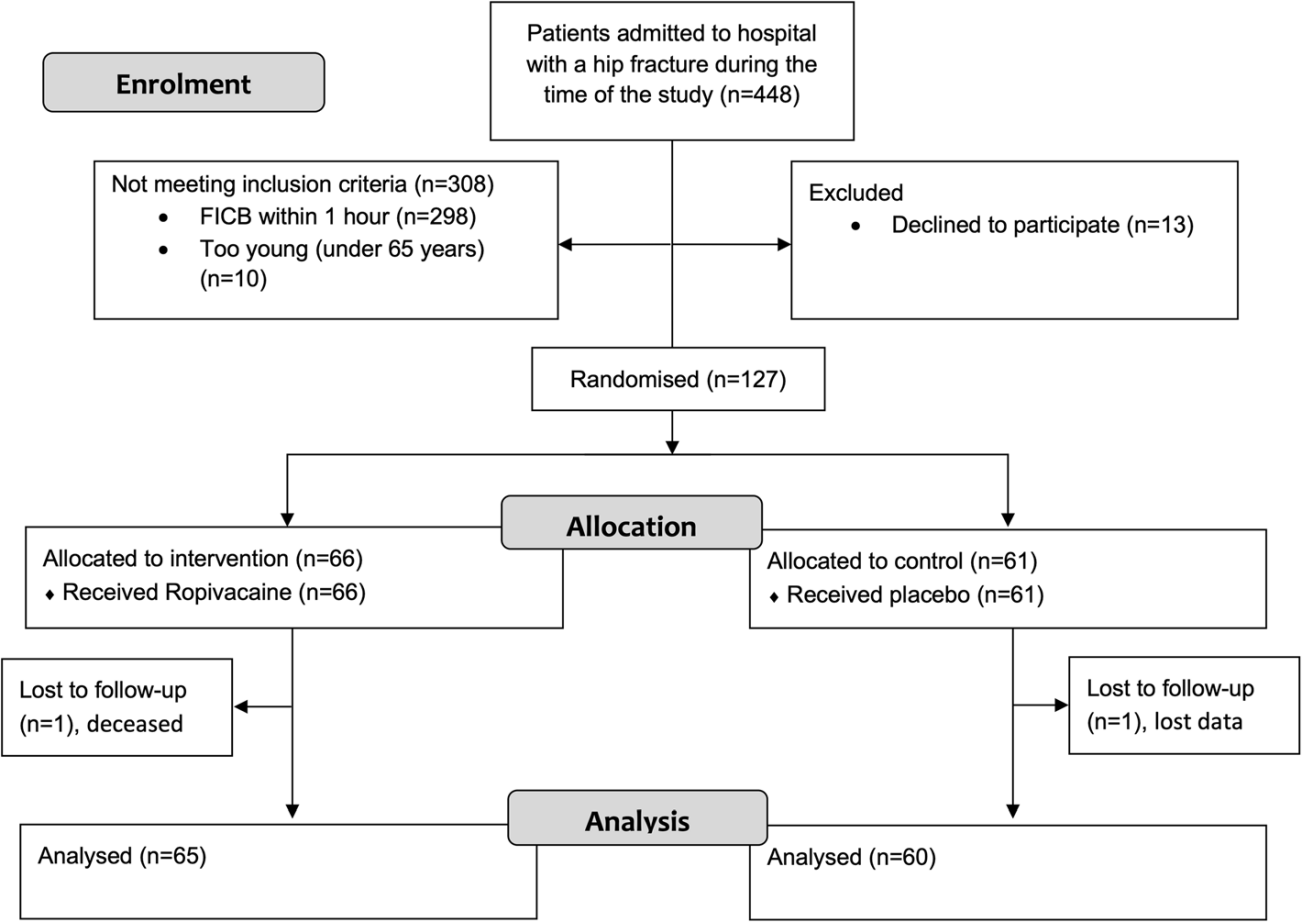
Flow diagram CONSORT 2010 showing the inclusion and analysis process of the trial

Patients with a suspected hip fracture were transported by ambulance directly to the department of radiology. After X-ray verification of hip fracture, the patients were transferred to an orthopaedic ward where those included in the study were randomised to either an intervention group or a control group. The FICB was performed in accordance with Dalen’s technique and administered as a complement to regular analgesia [28]. The medication used in the study was either 30 ml of ropivacaine 2 mg/ml (active substance) or 30 ml of isotonic saline (placebo). This dose was recommended by the Swedish Medical Products Agency. During office hours, the physician on the ward was contacted for study inclusion and FICB administration. After office hours, until midnight, the orthopaedic surgeon on duty was paged. All tests conducted on the study participants were carried out and registered in a case report form. A computer program was used for randomisation [29]. The randomisation (Fig. 1) and preparation of the study material were carried out by a statistics expert not involved in the study evaluation. The medication used for each individual patient was prepared by a nurse not otherwise involved in the collection of patient data.

All staff members were trained in using the pain assessment instrument, the Short Portable Mental Status Questionnaire (SPMSQ), filling out case report forms, randomisation and blinding according to the study protocol. All personnel involved in the study were blinded to the study medication. The study was conducted under the surveillance of an external monitor. Typical symptoms indicating adverse events (AE) associated with the injection of which the staff needed to be aware were hypotension, bradycardia, nausea, vomiting, paraesthesia, dizziness and headache.

### Regular analgesia

Morphine was given using an on-demand model with nurse-initiated analgesia. In Sweden, ambulances are staffed by prehospital emergency nurses (PENs). For PENs, it is standard procedure to titrate intravenous (iv) morphine for pain relief from the site of injury, during transport and until the time of admission to hospital. The titration of morphine was continued by nurses on the ward. Before surgery, paracetamol was prescribed on demand. This was a routine that was considered to work acceptably. There were no formal standards with regard to the preoperative dosage of either morphine or paracetamol. In hospital, preoperative iv morphine was usually prescribed as 1–2 or 2.5–5 mg on demand, repeated if necessary.

### FICB

The FICB was administered to the affected hip by a perpendicular injection with a two-pop technique as a complement to preoperative analgesia by the orthopaedic surgeon who examined the patient. The insertion point was identified by drawing a line between the *spina iliaca anterior superior* and *os pubis*, 1 cm lateral to the conjunction of the two thirds closest to the *spina iliaca anterior superior*. The insertion was made with a regular needle for intramuscular injections by loss of resistance when passing first the *fascia lata* and then the *fascia iliaca* (two pops). The investigation fluid was then injected [28]. Thirty-four physicians performed the FICB.

### Patient characteristic data

The following parameters, retrieved from the patients’ medical records, were used to characterise the study patients: age, gender, type of fracture, analgesia, pain, diagnosis of dementia prior to enrolment and the American Society of Anesthesiologists’ Physical Status Classification System (ASA). The ASA score system categorises comorbidity [30]. The first category (ASA 1) describes a physically healthy person and the fourth category (ASA 4) describes a person with considerable medical impairment. The fracture was defined according to the classification of hip fractures used by the Swedish National Registry of hip fracture patient care [31]. Fractures were classified as cervical, trochanteric or sub-trochanteric.

### Measurements

For the purpose of cognitive screening, the Short Portable Mental Status Questionnaire (SPMSQ) was used [32]. This is a 10-item questionnaire that can be administered orally and shows good sensitivity and specificity [33]. The SPMSQ is quick and easy to administer and well suited for screening large populations for cognitive status and severity of deficit [34]. The SPMSQ has been used in several studies [10, 20, 35, 36].

The SPMSQ consists of the following questions: 1. What date, month and year is it?; 2. What day of the week is it?; 3. What is the name of this place?; 4. What is your phone number?; 5. How old are you?; 6. When were you born?; 7. Who is the current prime minister?; 8. Who was the prime minister before him?; 9. What was your mother’s maiden name?; 10. Can you count backwards from 20 by 3’s? Every correct score gives one point. Mother’s maiden name is scored as correct if it is not the same as the patient’s surname.

The scores on the SPMSQ were divided into four groups 0–2; 3–5; 6–7; 8–10. The scores of 0–2 are regarded as severe cognitive impairment; 3–5 and 6–7 are regarded as moderately or mildly impaired and 8–10 is considered cognitively intact [19]. The SPMSQ does not identify delirium or dementia, but screening can help healthcare providers to take proper action when an impaired cognitive status is present.

The instrument used to assess pain was the Stockholm South General Hospital Pain Instrument (SSGHPI) [37]. The SSGHPI is a combination of three self-rating scales: a visual analogue scale, a numerical rating scale from 0 to 10 and a verbal rating scale. The patient used the scale that he or she found most appropriate from the three alternatives. The fourth scale, a behavioural rating scale (BRS), was used by the healthcare providers only when the patients were not able to assess and describe their own pain. The BRS is a three-category scale categorising pain from the patients’ behaviour. The three categories are: 1 – no pain or mild pain, 2 – moderate pain and 3 – severe pain [27].

### Data analysis

Descriptive statistics such as means, standard deviations (SD), median, range and proportions were used to summarise socio-demographic and clinical characteristics. For comparisons between the two groups with respect to categorical data, the chi-square test was used. When comparing groups with respect to morphine dose, we dealt with skewed distributions deviating from normal distribution, so we used non-parametric tests (the Mann-Whitney test). After establishing a classification of SPMSQ change from admission to postoperative period in three classes (decreased, increased and unchanged level), we also used the chi-square test to compare the distribution of the classified categories between the two groups.

A *p*-value of < 0.05 was regarded as a statistically significant result. All analyses were performed with the IBM SPSS version 22 statistical package.

## Results

One hundred and twenty-seven patients were randomised to either the intervention group (*n* = 66) or the control group (*n* = 61). The two study groups were well balanced, see the data presented in Table 1. There were two drop-outs. One patient died during follow-up due to heart failure and one patient had unregistered SPMSQ data during follow-up.

**Table 1:** Patient characteristics

Description	Intervention group (n = 66)	Control group (n = 61)	*p*-value
Age, years			0.84
mean (SD)	84.6 (6.7)	84.9 (7.7)	
median (min; max)	85 (68; 99)	86 (65; 97)	
Gender*, n (%)*			0.78
Female	45 (68.2)	43 (70.5)	
Male	21 (31.8)	18 (29.5)	
ASA score*, n (%)*			0.55
1	1 (1.5)	3 (4.9)	
2	30 (45.5)	27 (44.3)	
3 & 4	35 (53.0)	31 (50.8)	
Type of fracture*, n (%)*			0.88
Cervical	33 (50.0)	29 (47.5)	
Trochanteric	29 (43.9)	29 (47.5)	
Sub-trochanteric	4 (6.1)	3 (4.9)	
Prehospital analgesia, *n (%)*	56 (84.8)	51 (83.6)	0.66
Prehospital morphine in mg,			0.99
mean (SD)	6.2 (4.7)	5.7 (3.5)	
median (min; max)	5 (0; 25)	5 (0; 15)	
Diagnosis of dementia prior to enrolment, *n (%)*	23 (34.8)	26 (42.6)	0.37
Hours from FICB to second SPMSQ score, *mean (SD)*	33 (13)	33 (12)	0.696

### The impact of FICB on cognitive status

Cognition scores were obtained at hospital admission and on the first postoperative day. There was no difference between the intervention group and the control group regarding the distribution of patients in SPMSQ categories at hospital admission. Nor was there any difference in the distribution of patients in SPMSQ categories on the first postoperative day. Both groups had an increased proportion of patients in the SPMSQ 0–2 group (Table 2).

**Table 2:** Cognitive status on admission to hospital and on the first postoperative day

Group	SPMSQ category on admission to hospital (*n* = 127) (*p* = 0.6)				Postoperative SPMSQ category (*n* = 125) (*p* = 0.5)			
	0–2	3–5	6–7	8–10	0–2	3–5	6–7	8–10
Intervention, *n (%)*	14 (21)	7 (11)	7 (11)	38 (58)	19 (29)	2 (3)	12 (18)	32 (49)
Control, *n* (%)	11 (18)	9 (15)	10 (16)	31 (51)	15 (29)	4 (7)	7 (12)	34 (57)

*p*-value for distribution of SPMSQ scores between groups

The change in SPMSQ from arrival at hospital to the first postoperative day shows a positive, albeit not significant, trend in favour of the intervention group (Table 3). An increase in the SPMSQ score means an improvement in cognitive status, while a decrease in the SPMSQ score means a deterioration in cognitive status. More patients in the control group therefore showed a deteriorating cognitive status compared with the intervention group. Furthermore, more patients in the intervention group showed an improved cognitive status compared with the control group. However, the majority of patients showed an unchanged cognitive status.

**Table 3:** Change in SPMSQ category between groups from admission to first postoperative day (*n* = 125) (*p* = 0.3)

Group	Decrease	Unchanged	Increase
Intervention, *n (%)*	4 (6)	48 (74)	13 (20)
Control, *n (%)*	8 (13)	43 (72)	9 (15)

### The association between cognitive status and prehospital analgesics

Of a total of 127 patients, 110 patients received morphine in the ambulance. There was no significant difference between the proportion of patients receiving morphine in the ambulance in the different SPMSQ groups: *p* = 0.42.

There was no difference in the mean prehospital dose of morphine between the three groups, 8–10, 6–7 and 3–5. However, between the three groups and the 0–2 group, there was a significant difference (Table 4).

**Table 4:** Morphine doses administered in mg by SPMSQ group (*n* = 127)

Morphine administration	SPMSQ group			
	8–10	6–7	3–5	0–2
Prehospital	6.7 (4.7)	5.7 (3.3)	5.6 (3.4)	3.7 (2.2)
0-2 h	2.7 (2.9)	2.5 (2.1)	2.4 (1.8)	1.8 (2.1)
2-6 h	3.3 (3.7)	3.5 (2.7)	3.4 (2.3)	2.8 (2.9)

0–2 h = interval from hospital admission to two hours after FICB; 2–6 h = interval from two to six hours after FICB. Mean doses (SD) are shown

The mean prehospital dose of morphine was significantly lower among patients with severe cognitive impairment (SPMSQ 0–2) than among patients with higher levels of cognitive status. However, no difference was found between the subgroups according to SPMSQ regarding the dose of morphine that was given after arrival in hospital (Table 5).

**Table 5:** Morphine administered in mg by SPMSQ group (*n* = 127)

Morphine administration	SPMSQ on arrival to hospital	n	Morphine dose in mg		*p*-value
			Mean (SD)	Median (min-max)	
Prehospital	0–2	25	4.02 (2.37)	4.0 (0–9.5)	0.009
	3–10	102	6.43 (4.39)	6.75 (0–25)	
0 - 2 h	0–2	25	1.70 (1.82)	1.5 (0–6)	0.20
	3–10	102	2.38 (2.45)	2.00 (0–18)	
2 - 6 h	0–2	25	1.14 (1.55)	0 (0–5)	0.58
	3–10	102	0.95 (1.49)	0 (0–7)	

0–2 h = interval from hospital admission to two hours after FICB; 2–6 h = interval from two to six hours after FICB

No serious adverse events were reported as a result of the FICB during the study period.

## Discussion

The hypothesis that a preoperative nerve block after a hip fracture would improve cognitive status was not confirmed in this study. A report by Mouzopoulos et al. [24] shows that repeated FICB before and after hip fracture surgery reduced the risk of postoperative delirium. As a control, they used intramuscular injections of pethidine on demand, combined with intravenous paracetamol. Preoperatively, we used intravenous morphine injections on demand, combined with paracetamol for control. More effective preoperative pain relief in the control group in the present study may be one reason for the lack of differences in cognitive status, when compared with the pain relief routine in the control group in Mouzopoulos’ study. Furthermore, in the present study, the patients were given a single FICB injection, whereas Mouzopoulos et al. repeated their FICBs throughout the perioperative period, which might have reduced the risk of delirium in their intervention group. The findings reported by Mouzopoulos et al. are supported by a study describing an association between improved pain control and a reduced risk of delirium [23].

Another reason may be a fading FICB effect when the waiting time (median 19 h) to operation exceeds the time window for the effect of the FICB. The effect of fascia iliaca compartment block has been shown to fade after eight hours [38]. We therefore hypothesise that FICB should be repeated throughout the acute phase in order to improve cognitive status in patients with hip fractures. One suggestion is that FICB should be administered every 12 h preoperatively. However, this hypothesis needs to be confirmed in a randomised clinical trial.

We found that patients with a cognitive impairment received lower doses of morphine before arrival in hospital. Previous researchers have reported similar findings. Patients with dementia received only one third to half of the morphine dose that cognitively intact patients received [15, 16]. Cognitive impairment has thus been reported to be the most common barrier to receiving adequate analgesia [39], despite the fact that cognitively impaired patients experience equal or maybe even higher levels of pain [40]. Difficulties in communication and a higher degree of comorbidity in patients with a cognitive impairment are probably some of the reasons for this phenomenon. This calls for careful dose titration by healthcare providers.

It is only possible to speculate on the reason for the discrimination of patients with a cognitive impairment regarding relief of pain. It seems reasonable to assume that, when titrating morphine, nurses have difficulty assessing the level of pain in cognitively impaired patients. Sometimes nurses need to rely on their own assessment of the patients’ pain [41]. The nurse is quite often alone with this decision. When healthcare providers feel insecure about patients’ pain levels, they may hesitate to decide on pain relief [42, 43]. Nurse-initiated analgesia through morphine titration on demand is a skill that is acquired through experience. The prescribed dosage often has an interval (for example, 2.5–5 mg of morphine iv on demand). The dose can be repeated, if necessary, after evaluation. This method requires time, knowledge, presence and dedication on the part of the administering personnel [44]. It is also possible that a more severe cognitive impairment requires more time for pain assessment by the nurse and lack of time is common in the everyday healthcare situation [45]. The frequencies of dosage varied between patients, which resulted in various total doses, visible when examining min-max variances in morphine administration. It is likely that the challenging pain assessment of the cognitively impaired patients’ pain and need affected the total morphine dose that was administered. Based on the reduced doses of morphine that the patients with the most marked cognitive impairment received before arrival in hospital, it is an attractive hypothesis that severely cognitively impaired patients have even more to gain from FICB than their lucid counterparts. If nothing else, it should be a requirement that all patients with hip fractures should receive at least 5 mg of morphine iv during their first hour after contact with a healthcare provider [45].

In spite of this, the ambulance nurses in this study were relatively liberal with the administration of morphine compared with other studies evaluating nurse-initiated analgesia [46–49]. It thus appears that cognitive status affects the dose of morphine that is given more than the morphine dose affects cognitive status.

The results highlight the need for more finely developed instruments for behavioural pain assessment and the need for more studies evaluating pain and its impact on patients with a cognitive impairment. For the future development of healthcare, this study is a simple reminder that the evaluation of pain is a complex and underestimated problem. Pain assessment, interventions for pain control and awareness of undertreated pain need to be improved, especially in emergency healthcare.

If FICB is to be implemented with the intention of improving pain control, its high level of safety is supported by this study, due to the absence of serious adverse events.

### Limitations

This study may have been underpowered in order adequately to address its primary aim. The main purpose of the original study was to examine pain control through FICB and sample size calculation was therefore performed using data on pain with a calculated power of 90% [27]. The present results in this paper show a 6% deterioration in SPMSQ score in the intervention group compared with a 13% deterioration in the control group. The results of this study of cognition should therefore be regarded as hypothesis generating rather than hypothesis confirming.

We evaluated cognitive function according to the SPMSQ. However, it would have been an advantage to have registered the incidence and prevalence of delirium in addition to SPMSQ scores. The SPMSQ score does not differentiate between delirium and/or dementia and it only shows the present status of a sometimes very fluctuating condition.

The interval from hospital admission to the first postoperative day differed between individuals. However, the mean delay did not differ significantly between the two groups.

Patients with displaced fractures are more likely to have severe pain. In this study, no data on displaced fractures were collected. It was therefore not possible to adjust the results for this potential confounder, which is a limitation.

Finally, patients were only included in the study during daytime and until midnight. Although this may have created a selection bias, a bias of this kind could hardly affect the results in terms of pain relief.

## Conclusion

We found no impact on cognitive status by a single FICB after a hip fracture. Patients with a severe cognitive impairment received less pain medication before arrival in hospital than their lucid counterparts. This discrimination needs to be further addressed. Cognitively impaired patients in particular may benefit from improved pain control and quality of care with FICB, but this remains to be demonstrated.

## Data Availability

The datasets used and/or analysed during the current study are available from the corresponding author on reasonable request.

## Abbreviations

ASA: American Society of Anesthesiologists Physical Status Classification System
FICB: Fascia Iliaca Compartment Block
SPMSQ: Short Portable Mental Status Questionnaire
